# Navigating complexity: understanding the experiences, motivations and challenges of conducting research with perinatal individuals who use opioids and other substances

**DOI:** 10.1186/s12884-026-09017-0

**Published:** 2026-06-08

**Authors:** Renee C. Edwards, Seema K. Shah, Brianna K. Sinche, Rachel Ahrenholtz, Quyen Cao, Tabitha J. Davis, Abigail L. Blum, Ashish Premkumar, Elizabeth S. Norton, Suena H. Massey, Lauren S. Wakschlag

**Affiliations:** 1 Northwestern University Institute for Innovations in Developmental Sciences, 625 N. Michigan Ave, Suite 2400, Chicago, IL 60611 USA; 2 Department of Medical Social Sciences, Northwestern University Feinberg School of Medicine, 625 N. Michigan Ave, Suite 2100, Chicago, IL 60611 USA; 3 Department of Pediatrics, Northwestern University Feinberg School of Medicine, 225 E. Chicago Avenue, Box 86, Chicago, IL 60611 USA; 4 Lurie Children’s Hospital, 225 E. Chicago Ave, Chicago, IL 60611 USA; 5 Department of Psychology, University of Central Florida, Building 99, 4111 Pictor Lane, Orlando, FL 32816 USA; 6 Boston Children’s Hospital, 300 Longwood Ave, Boston, MA 02115 USA; 7 Department of Psychology and Human Development, Vanderbilt University, 2301 Vanderbilt Place, Nashville, TN 37240 USA; 8 Department of Obstetrics and Gynecology, Section of Maternal Fetal Medicine, Biological Sciences Division at the University of Chicago, 5841 South Maryland Ave, Chicago, IL 60637 USA; 9 Roxelyn and Richard Pepper Department of Communication Sciences and Disorders, Northwestern University, 2240 Campus Drive, Evanston, IL 60208 USA; 10 Department of Psychiatry, Massachusetts General Hospital, 185 Cambridge Street, Boston, MA 02114 USA

**Keywords:** Opioid use, Perinatal substance use, Recruitment, Retention, HBCD

## Abstract

**Background:**

Despite increased opioid use (OU) among individuals of child-bearing age, pregnant and postpartum individuals with OU, and the experiences of these individuals and scientists with related expertise, are notably absent in scientific literature and discourse. The HEALthy Brain and Child Development (HBCD) Study, a large, longitudinal, U.S.-based consortium project, aims to understand how brain and child development is shaped by various adversities and protective factors, including prenatal exposure to opioids and other substances. Given the persistent challenges of engaging pregnant and postpartum individuals with OU in longitudinal research, this qualitative study, conducted during the pilot phase of HBCD, aimed to better understand barriers, motivators, and best practices to recruitment and retention of this underrepresented population.

**Methods:**

Participants included perinatal individuals with opioid use disorder (OUD) (*n* = 6) and researchers with expertise in perinatal substance use (*n* = 22). Semi-structured interviews were conducted and thematically analyzed to identify effective strategies for recruitment, engagement, and retention in longitudinal research. Knowledge and comfort with neurodevelopmental research procedures was also explored.

**Results:**

Key themes emerging from both participant groups included the centrality of trust and nonjudgmental relationships between research teams and participants, transparency about research aims and procedures, and meaningful connections to emotional, educational, and logistical supports. Respondents emphasized the value of research staff continuity, flexible study designs, and opportunities for participants to share their stories and receive feedback. Although participants generally expressed comfort with infant neuroimaging and biospecimen collection, misunderstandings about these methods underscored the need for thorough, accessible education.

**Conclusions:**

Findings highlight the importance of responsive, ethically grounded research practices to strengthen research on opioid use in a manner responsive to the needs of affected populations. Successful engagement requires creative and adaptable study designs, ancillary supports, and research staff who foster trusting relationships and help participants feel heard and valued.

**Supplementary Information:**

The online version contains supplementary material available at 10.1186/s12884-026-09017-0.

## Introduction

Despite widespread efforts to regulate the supply of opioids in the U.S. in recent decades, the opioid epidemic continues to affect millions of families [1]. Since the epidemic includes females of child-bearing age, increases in maternal opioid-related diagnoses—from 3.5 mothers per 1000 deliveries in 2010 to 8.2 per 1000 deliveries in 2017—have mirrored nationwide trends [2, 3]. Studies on prenatal opioid exposure have found that there may be risks to children’s neurodevelopment, including cognitive impairments, nervous system activity, and behavioral problems (e.g., [4–9]), but nuances related to timing, dosage, and polysubstance use, and importantly, protective factors that may buffer against these negative effects, are less well understood. Thus, further investigation is needed to elucidate potential impacts more comprehensively and inform interventions for affected children and families.

Inclusion and retention of participants using opioids and/or other substances during pregnancy in longitudinal research poses practical and ethical challenges. These individuals often face considerable stigma and may fear judgment or bias from research teams [10–12]. Many experience psychological challenges, including trauma histories, interpersonal violence, and mental health issues [13, 14] and logistical barriers, such as housing instability and lack of transportation, which can hinder participation. Legal concerns, since prenatal substance use (SU) is criminalized in some states and may lead to carceral involvement [15], and involvement from child protective services can further deter participation [16]. Recruiting individuals with opioid use (OU) specifically may pose additional challenges, as prenatal use is especially stigmatized [17], and many women receiving medications for opioid use disorders (MOUD) have daily clinic visits [18] that may limit their availability to participate in research visits elsewhere.

Additionally, a rigorous and comprehensive understanding of the impact of substance exposure on maternal and child health is greatly strengthened by integrating diverse and complementary data collection methods, such as biospecimen collection, neuroimaging (e.g. EEG, MRI), physical assessments, direct child and family observation, neurodevelopmental assessments, and surveys [19, 20]. This comprehensive approach can pose substantial burden for families, given the time required to complete visits, the need for frequent follow-up visits, and the wide range, unfamiliarity and intensity of study activities. Participants may also have safety concerns and apprehension of findings that might emerge from neuroimaging, biospecimen, and developmental assessment measures [10, 11, 21]. For a population already hesitant to participate in research, these “asks” underscore the importance of thoughtful engagement strategies informed by lived experience.

Prior research shows that longitudinal studies with strong recruitment and retention rely on strategies such as flexible scheduling and visit locations, staff continuity, community involvement, frequent check-ins, and material support [22, 23]. Recruitment of individuals with SU may be further strengthened through resonant messaging (e.g., altruism, giving back to the community), accessible study materials, monetary incentives, and outreach to treatment and recovery programs [11, 24, 25]. For pregnant participants and families, access to medical services (e.g., ultrasounds), baby-focused gifts, broad evaluation windows, transportation, childcare, and supportive services can enhance participation [10, 11]. Yet, given the persistent challenges of recruiting and retaining perinatal individuals with OU, it is critical to center their voices to better understand barriers and facilitators to their engagement, alongside insights from experienced researchers.

The HEALthy Brain and Child Development (HBCD) Study [26] (https://hbcdstudy.org) is a national, longitudinal consortium study that aims to map typical neurodevelopmental trajectories starting at birth and examine how prenatal exposure to opioids and other substances, as well as various environmental, social, and biological factors, impact brain and child development. HBCD is enrolling over 7,000 pregnant individuals at research sites across the U.S. and aims to oversample for prenatal SU (~ 25% of the sample) and OU specifically (~ 12% of the sample). This qualitative study was part of Phase I of the HBCD study (preparatory work preceding the enrollment of participants into a national cohort) and was designed to inform best practices for optimizing engagement and retention of perinatal individuals with OU. By including both perinatal individuals with opioid use disorder (OUD) and researchers experienced in studies of perinatal populations who use substances, this project uniquely enabled comparison of diverse perspectives on research engagement.

## Methods

### Recruitment

#### Patients

A sample of pregnant and postpartum individuals with OUD (“patients”) were recruited from (a) inpatient admissions for management of OUD during pregnancy at a large urban hospital in the midwestern US, (b) a previous study that enrolled a similar population in a large midwestern city, and (c) direct referral from an obstetric care provider specializing in perinatal substance use at a large urban hospital. Individuals were eligible if they were at least 18 years old, spoke English, were pregnant or within 8 weeks postpartum, and had a diagnosis of OUD during pregnancy. Recruitment of patients through sources (a) and (b) proved challenging due to an inability to reach and/or schedule patients for an interview, so the team pivoted to recruit primarily through the third approach.

#### Researchers

A purposive sample of researchers with expertise conducting studies with perinatal individuals with SU (“researchers”) was recruited. Individuals were initially identified from an established group of researchers with content expertise and from the study team’s professional network. Subsequently, researchers were identified through snowball sampling. To be eligible, researchers needed to speak English and have experience conducting research with perinatal individuals with SU (including but not limited to OU).

### Interview guides

Participants were interviewed using semi-structured interview guides covering relevant topics. Creation of these guides was informed by literature reviews, the overall HBCD Study goals, and input from an interdisciplinary team of HBCD consortium investigators. A brief demographic survey followed each interview.

#### Patients

The interview guide for perinatal participants included questions regarding their experience with and views on research; perceptions and trust of researchers and prenatal care providers; facilitators, barriers, and motivators to research participation; recruitment sources and strategies; and knowledge and comfort participating in neuroimaging (infant EEG, MRI) and providing biospecimens (maternal and infant blood, saliva, hair, nails) as part of research (Appendix A).

#### Researchers

The interview guide for researchers focused on their experiences conducting research with pregnant and parenting individuals with SU, approaches to enhance participant comfort, and approaches they advise against (Appendix B). Additional questions regarding laws governing prenatal SU and experience with the justice system and/or child protective services are reported elsewhere [12].

### Interviews

Study procedures were approved by the Institutional Review Boards (IRBs) at Washington University (St. Louis, MO), Northwestern University (Chicago, IL), and Lurie Children’s Hospital (Chicago, IL). The research was conducted in accordance with U.S. federal regulations (45 CFR 46, the Common Rule) and ethical principles described in the Belmont Report and the Declaration of Helsinki. Informed consent was conducted by telephone; written consent forms were provided electronically over a secure server. All participants were interviewed by phone or through video conferencing by female researchers with whom they had no prior interactions. Interviewers were research coordinators or managers (including the third and fourth authors) who had several years of research experience with vulnerable populations, were trained in nonjudgmental interviewing, and received regular supervision by the lead investigators (first and second authors). Video calls were password protected, with interviews conducted in private locations. Audio recordings were saved on a secure server within password-protected folders. Interviews were conducted between October 2020—September 2022 and lasted 30–90 min. Participants were given a $50 Visa gift card.

### Interview analysis

A qualitative descriptive design was chosen to provide a rich, contextual description of participants’ perspectives on engagement in longitudinal research. Qualitative descriptive approaches are well-suited for applied health research when the aim is to describe participants’ experiences in their own words without imposing a highly interpretive or theory-generating framework [27, 28]. This design allowed for open-ended responses using a semi-structured interview guide and emphasized staying close to the data to generate findings that are directly translatable to research design and engagement strategies.

The patient interview recordings were transcribed verbatim by a research assistant, and transcripts from the researcher interviews were generated through video conferencing software and reviewed for accuracy by a research coordinator. Transcripts were deidentified before uploading to a qualitative analysis software program (Dedoose, version 9.0.107, 2021). Patient interviews were coded and analyzed by the first and third authors, and researcher interviews by the second author and two masters-level coordinators. Initial codebooks for each sample were developed based on the questions and themes addressed in the interview guide. Researchers then independently coded the first transcript in their sample and subsequently met to review the transcript and initial codes and identify new codes based on participant responses. The codebooks were revised and added to through discussion after the first three transcripts in each sample, until no new codes emerged (see Table 1). All remaining transcripts were double-coded and teams met to resolve any disagreements. Participants did not review transcripts or codes.

**Table 1:** Final codebook

**Patients**	**Researchers**^a^
Barriers to research participation	Recruitment and engagement
Connections to resources and support	Communication about study goals
Facilitators for research participation	Relationship-building, trust, nonjudgmental
Knowledge of substance use (SU) laws	Connections to resources, services, support
Motivations for research	Staff continuity
Openness with providers about SU	Creative solutions to barriers and minimizing burdens
Perceptions of biospecimen collection	Consenting
Perceptions of neuroimaging	
Perceptions of research/researchers	
Pregnancy experience	
Prenatal care	
Recruitment sources	
SU (including stigma)	
Trust in providers/researchers	

^a^Additional codes related to legal/regulatory issues are reported elsewhere [12]

After all transcripts from both samples were coded, thematic analysis was conducted [29]. The first and third authors identified main themes by reviewing the coded text and examining patterns in the responses. They met to discuss and come to consensus on themes and select participant quotes that most effectively represented the themes. A second phase of analysis was later conducted to identify cross-cutting themes through an integrated review of coded excerpts from both patient and researcher interviews. Empirical evidence suggests that 6–17 interviews are often sufficient to reach information power in qualitative studies [30], particularly when samples are relatively homogenous and research aims are narrow and focused [30, 31]. In the present study, thematic patterns were apparent in the early interviews and subsequent interviews primarily elaborated and refined existing themes. This likely reflects the structured interview guide and focused inclusion criteria (perinatal individuals in treatment for OUD).

The current study conformed to COREQ criteria (Appendix C). Credibility and dependability were enhanced through use of a structured open-ended interview guide, standardized interviewing procedures, a systematic coding process with coding meetings after each interview to ensure consistent application of codes, and team-based analysis that prioritized participants’ own language and meanings. Transferability was facilitated by detailed description of participant characteristics and research context.

## Results

### Sample demographics

#### Patients

Six individuals with a diagnosis of OUD during pregnancy were referred, provided consent and participated in an interview. Four resided at a substance use treatment facility and two were interviewed while at home. The average age was 27.8 years, all were female and all unmarried. They represented various racial (White and Black), ethnic (Hispanic and non-Hispanic), and educational (middle school, high school, GED) backgrounds. Due to the small sample size, exact frequencies are unreported.

#### Researchers

Forty-three researchers were contacted about the study, and 22 were eligible, provided consent, and participated. Most participants were female (*n* = 17), the majority were non-Hispanic White (*n* = 19), and the average age was 52.3 years. The academic disciplines represented included clinical psychology (*n* = 9), medicine (*n* = 9), and epidemiology/public health (*n* = 4), all had advanced degrees, and most conducted research across a mix of geographical settings. Over half (*n* = 10) had more than 20 years of research experience.

### Thematic analysis

#### Common themes

Four common themes emerged from both the patient and researcher interviews: (1) centrality of trust; (2) transparency throughout the research process; (3) long-term commitment; and (4) meaningful connections to resources.

##### Theme 1: centrality of trust

Concerns about being judged for OU were pervasive in the patient interviews. All six described fear of judgment as their main hesitation with research, recounting experiences of shame and dismissal by medical providers, family, and friends. Most recounted their hesitancy to disclose their SU to prenatal providers or even seek prenatal care; however, they all eventually shared this information to get the best help for themselves and their infants.

I wanted to be open and honest with [my providers] about everything, so that way my baby could be taken care of, to the best of their ability. At first…[m]y level was just like very, very, shy. I didn't wanna be judged, but then after I became comfortable, I allowed myself to be open and honest about everything. They didn't seem to judge me. They didn't get into the whole, “oh, you're bad, you're a bad mom for doing that, you shouldn't have done that” and it wasn't judgment at all. They were just very, very helpful, very understanding, like “it's okay, you're gonna get through it. We're proud of you for taking the steps you've taken.” (P2).

As this participant suggested, trust was developed when providers validated their feelings and followed through with connections to services. Other comments about developing trust with researchers highlighted features of researchers that were helpful for building trust.

Somebody that's just open-minded and very reassuring and sensitive where they're acknowledging everything that we're talking about and they understand, they get it. Instead of just being there, staring at you talking. So we're not feeling so judged while we're telling our stories to strangers, you know? It's hard for moms to wanna come forward and talk about the experiences that they're having without feeling guilt and choking up … they're realizing what they did is, it's hard for them to talk about. (P3)

Additionally, prior positive experiences of developing trust in researchers made a difference:

…the researchers that I've had, they've been very open and honest…and they were sensitive because they did mention that you don't have to talk about something if it's hard for you to talk about… And everything that I was saying did matter to them and it was all noticed…So it made me feel like I was making a difference and they were in it for the best interest. (P3)

Similarly, researchers stressed the importance of developing trust by being supportive, non-judgmental, genuine, and caring. Several emphasized that stigma from negative societal perceptions of prenatal SU makes it essential to create an environment where participants feel valued rather than judged. Trust-building strategies ranged from smaller gestures (e.g., not wearing white coats) to larger efforts (e.g., hosting celebrations for families). One researcher emphasized the mistrust that many individuals feel about research, often stemming from negative experiences with healthcare and other institutions. Researchers felt they must invest time and effort to gain participants’ confidence by being supportive and understanding of their unique situations.

When we talk to these women in the hospital, a lot of them as soon as you mention the word research, ‘no, no I don’t want to’ and they don’t trust the system. And they have good reason not to trust the system. Ok, so you’ve gotta win their confidence. You have to be supportive, non-judgmental, show them you care. (R5)

Make sure they know you’re doing it because you care about them and you want to make their world better and they’re helping you by letting you in. (R19)

Staff training was also seen as essential to help the team develop and maintain a sensitive approach. For example, one researcher expressed the importance of:

Being very flexible, non-judgmental and understanding with your participants and training your staff to be as well. Making sure your staff understands that substance use while pregnant is not something people choose. It is not a moral failing. It’s not a sign of being a bad mother. There’s a wide range of how that happens, when it happens, and that nobody’s actually a perfect mother anyway, and people are trying really hard. (R20)

Patients described wanting to feel like more than just a number or data point. They wanted to share their experiences deeply, even when research questions do not explicitly probe, and they valued research teams who listen attentively. For example, one patient noted approaches that could erode trust:

When you get to talking about something that, you know, could be on more of a deeper level, and then it almost feels like you're being cut off or the questions be completely switched to something else and then, you know, whatever it was that you got so deep into is not really that important. That right there kind of makes you feel like it’s just a test on me…and things like that can make me feel really discouraged. (P1)

Most patients did not consider researchers’ backgrounds to be important for establishing trust, though one mentioned that staff who share the same ethnicity, are female, and from the same generation could be helpful. Another suggested that hiring someone with their own lived experience of OUD, such as a peer support specialist, and ideally a mom, would be helpful to build trust. There was also mention of child-friendly staff. *“The only thing that would make me comfortable is somebody that's really the ‘kid person’… I would want them to really try to make my son feel open…a fun person.”* (P3).

While several patients did not believe their culture or family background impacted their views, one shared that her family was distrustful of research, and that culture, race, and education can impact people’s perspectives. *“…I’m not trying to sound racist, but I come from a background where we don’t do research.”* (P4) No one she knew had participated in research, but she joined this study to make her voice heard and improve things for other pregnant women using substances and working on recovery.

Trust also emerged as a key aspect of study recruitment. Patients overwhelmingly felt the best recruitment sources are trusted medical providers or social workers who can vouch for the study. *“That would be great because it's someone [prenatal care provider] that, at least with me, I have grown to trust.”* (P1) They also suggested approaching mothers in prenatal clinics, public aid offices, neonatal intensive care units and substance use treatment centers. Researchers noted the need for discretion and respect during recruitment, recommending partnerships with clinics to ensure sensitive, appropriate recruitment methods.

If you’re doing something that might put people at risk of harm or stigma, that’s not cool. I may be very interested in the population that is served by the methadone clinic, but instead of standing outside and putting flyers on people’s cars, I would try to interact with the administrators of the clinic and, you know, figure out a more appropriate and discreet way to advertise my study. (R1)

To address trust during recruitment, some researchers hired staff who are familiar with clinics and the healthcare system, collaborated with clinic staff, and identified a ‘clinic champion’ whom women trust to promote the study.

Building connections with clinic staff where you are is very important. A lot of people we hire are from within the health system, so they have experience…and in having that personal contact and knowledge of how the clinic works and of the clinic staff, either that they’ve had coming in or have built, those being part of the study is critical. (R6)

I think trust building is a huge thing in these types of studies. How do you connect with, how do you identify with that one champion within your stakeholder group that can help you build that trust. Because even though you may go in as a researcher that is really being honest and open, they’ve been scarred so much that they may not be willing to talk to you. (R3)

Trust was also a factor in patient views on social media recruitment – some saw it as an effective way to spread information, while others viewed it as untrustworthy or akin to telemarketing.

I do see a lot of TikTok videos and there's pediatrics on there and nurses that are OB-GYNs that I follow and get parenting classes from. They do short videos about it and it's easier for me to be more interested and I'm like, oh, I'm intrigued by that, and I go and click on the link. (P3)

Some people don’t be interested [in researchers “cold calling”], some people just say, telemarketer… The doctor’s office is better because they can just tell them right then and there what the study’s about. They would think that [social media advertisements] is telemarketing as well. (P4)

##### Theme 2: transparency

Patients and researchers both stressed the importance of transparency throughout the research process, including study purposes, risks, benefits, and results. Patients want ongoing updates throughout the study, and for researchers to “*be real about it*” and not make it seem like “*the greatest thing in the world.*” In other words, they felt that researchers should not over-promote the study or exaggerate findings.

I would just want to know the whole thing about what we are going to do, what is going to be said, what we are going to go over, how this is going to help, like, literally everything. (P6)

I'm all for helping research and helping other women with you know, helping doctors who can treat pregnant women with opioid addiction better, but I don't want to do anything that might risk me and my daughter's health or could cause any problems…I just want to know all the information. (P5)

Overall, patients want assurance that meaningful knowledge is being generated from the study. They are interested in hearing other participants’ stories, learning about positive research outcomes, and understanding the implications of less favorable results.

Researchers similarly stressed the importance of clarifying study goals, procedures, and the reasoning behind survey questions; conveying how valuable participants’ contributions are for advancing knowledge; and giving participants autonomy to make decisions about different study activities, connecting this to concerns about stigma by noting it is a way for participants to help combat stigma.

I think that these women experience an enormous amount of stigma and judgment from society and they need to know that’re not just guinea pigs and that the information they can provide is extremely valuable and helping us…So when we talk about the importance of the science and how it contributes to the development of the baby, I think it’s a really strong pull for them to want to participate in this kind of research. (R2)

One researcher suggested openly discussing mandated reporting requirements and providing concrete examples, especially around SU and child maltreatment, to promote transparency, based on prior community consultation.

I now explain very clearly what is reportable and what isn’t to participants, which I actually do because of feedback from a community partner (a reproductive justice group and advocacy group). They thought the way my consent was written, where I said what I had to report, wasn’t really clear…like what is child abuse, what would be called child abuse? I was like ‘good point’…[now] I actually give them some examples of what is reportable and what is not reportable about their use in the home with children. (R20)

Some researchers highlighted that transparency should extend to the results and dissemination phases of research to show participants what happens with collected data. For example, one researcher suggested a useful exercise in which researchers imagine participants reading their publications and reflect on how participants might understand and react to the information presented.

…would you be worried about how they’re going to respond or do you think they’d be surprised to learn that is what their participation was for? I wouldn’t want a participant to read a paper that I publish and think “I had no idea that’s what the data were for.” (R7)

##### Theme 3: long-term commitment

Both participant groups stressed that study retention depends on having research staff who are nonjudgmental, empathetic, and caring. Patients suggested intermittent phone calls from the research team to maintain their interest in the study, along with continuous feedback about the study and outcomes, such as through newsletters and opportunities for discussion. They would want to know in advance what the next research visit will entail. One patient acknowledged that she would struggle with follow-up and long-term engagement, so it would take extra effort from researchers to maintain her participation. Several patients mentioned continuity in staff over time so trust can be developed.

Researchers similarly felt that continuity in trusted staff helps participants feel comfortable and remain involved.

[W]e try to make sure that once somebody agreed to participate, we had the same research assistant following that woman throughout the pregnancy. So they have a rapport, they know us, they can call us if they went into labor. (R12)

…our study went on for twenty years and we had the same social worker the entire time. Other staff changed as the kids got older, but I think that made a huge difference, she was a constant. She knew everyone and was constantly on the phone. (R22)

One researcher even described using the same driver throughout a study:

Transportation was an issue and we got to know one cab driver and he became our cab driver… He knew the families and they knew him…when we would have celebrations he’d become part of our celebration…we found that if we sent the cab driver that worked better than saying to the family, ‘get a cab and come here’ if they didn’t have a car. So that ended up being a really good thing for us for years. (R14)

Celebrating special events, birthdays and holidays with families was also mentioned several times. Researchers felt that for many participants, these moments of celebration might be rare outside the research context, making such gestures even more meaningful.

[F]or us, the child coming in with the mother was an opportunity to celebrate. We would celebrate the kid, we would celebrate the mom, the family, you know again and again. This didn’t happen for these families other than with us. You know, on birthdays we gave them birthday cakes. Thanksgiving, Christmas, we gave them turkeys. I mean, we did a lot of stuff. And we found excuses to help them that you wouldn’t normally do, because they were so needy. (R5)

Finally, researchers advocated for flexibility in meeting participants’ needs and minimizing small burdens that could add up over time, such as coordinating research visits with medical appointments, collaborating with clinic staff for sample collection, providing cash compensation, and offering flexible scheduling and weekend visits. If a participant did want to withdraw from a study, one researcher discussed the importance of first listening and clarifying any misunderstandings, while recognizing that they must ultimately respect participants’ decisions.

##### Theme 4: meaningful connections to resources

Several patients suggested that studies should provide referrals for counseling or even have a counselor on the research team. One noted that women using substances may be afraid to seek counseling or have uncertainty of where to look or just need that extra “push.” They also wanted information on parenting and local parenting classes and felt it would be helpful for researchers to facilitate connections to services. Most agreed that child development information at different ages would be especially useful, particularly if there are opportunities for discussions with researchers.

I would want to talk with someone because I want to know what’s going on with my child…if I read a piece of paper, I probably won’t understand. But by talking with someone, I’ll probably understand more, or I’ll try to understand more. (P4)

Transportation, childcare, and financial compensation were also mentioned as important resources.

Yeah, basically [I participate] if they pay for it. That sounds horrible, but all the surveys and stuff that I've done throughout the pregnancy, they always pay me for them … If it's just someone reaching out to talk and stuff, I was like ‘no,’ cause I have a lot of people that reached out to me already. (P5)

Researchers similarly emphasized providing meaningful connections with services along with warm handoffs, especially with treatment programs [32]. Referrals without proper support can feel like a token gesture but helping participants get access to services demonstrates a more compassionate approach to research.

I think when substance use in pregnancy is getting looked at as part of research, there’s obviously trust issues that are going to be huge. But I think [you need] a significant collaboration with genuine and real ways to help people in treatment, not just handing a card and saying ‘here’s a referral’ to satisfy the IRB.… I think treatment programs for ongoing substance issues unfortunately often exclude pregnant women, so constructing a study where there’s real and significant linkages with treatment programs, not in a punitive way, but that are genuinely offered and there’s real clinical expertise and trying to help women get help. (R15)

###### Additional patient themes

Two additional themes emerged from the patient interviews but were not prominent in researcher interviews: (5) altruism; and (6) comfort with study procedures.

##### Theme 5: altruism

Most patients shared primarily altruistic motivations for participating in research on OU. They valued opportunities to help other women, break the chain of SU in their family and community, and satisfy their own curiosity. Several noted a lack of information on the impact of OU on children and wanted researchers to study it and provide support and resources to women in recovery. They hoped that sharing their experiences would allow them to gain knowledge from research findings.

I want to make things better for people like me. Especially pregnant women who’s under the influence and, you know, going through what I’m going through and recover…I want to do as much research as I can so I can help other people out. (P4)

When somebody’s asking me to do research, it’s like, yeah, if it starts with me to help the future, the next generation, I’m all for it. (P3)

Another shared that seeing researchers really want to help can actually “*do wonders for one’s own recovery*” and she “*would love to see someone else benefit from this.*” Although one patient did acknowledge that payment was her primary motivation for participating, this particular patient was skeptical of researchers who emphasize money as a motivation:

But at the same time, there are a lot of times where you see people saying, “oh, we're doing research on this, we're doing research on this and that. Do you wanna be part of this, or be part of that?” And then the first thing they say, you know, “we give you money, so yada yada yada.” (P1)

##### Theme 6: comfort with study procedures

Patients also shared their perspectives on specific procedures, study duration, and visit locations that could be involved in longitudinal research. One said she would join a study lasting a few weeks but not one lasting a few months, while others felt a couple visits a year over several years was reasonable. Participants expressed mixed feelings about remote versus in-person research visits. Some felt that remote visits could be more convenient but also less personal, while others expressed privacy concerns.

[Remote visits] should be offered because … to have to make those trips, whether it's like one or two a year, but still making those trips could end up being very inconvenient, especially someone with a child or if they're working or whatever the case is so it might be a huge relief…but I think it's also a lot easier to kinda blow off at the same time. And I think you lose the personal side as well. (P1)

Coming from a mom who has kids and is doing substance abuse, it's kind of like a meeting, an AA meeting. We don't wanna do AA meetings online. When they were doing Zoom meetings for AA, I was not joining because it's not that confidential, because we can't really open up and talk about what we wanna talk about. Because we have our family members in the background and they're listening and we're ashamed…So I feel like [research] is just a little bit more personal and it gets people to open up more if they're in person. (P3)

Patients generally agreed that home-based research visits offer greater convenience but less privacy if family members are home. One patient further noted that home visits would be infeasible while residing in a treatment facility.

Overall, there were few concerns about infant neuroimaging and biospecimen collection (saliva, nails, hair, maternal blood). All participants had heard of MRI but also believed that MRI involves exposure to radiation, though most still felt it was safe. After the interviewer explained the procedure and its safety (see Appendix A), all agreed they would allow their infant to receive a “lullaby” MRI (natural sleep MRI without sedation). Participants were less familiar with EEG, but after hearing the description, none expressed any concerns. They were generally comfortable providing biospecimens for themselves and their infants, with a few caveats. Several would want to check all procedures with their doctor first, one felt that collecting baby’s hair and nails “*goes too far*,” and another was comfortable as long as neither she nor her child were asked to take medications.

The only thing that makes me uncomfortable is I don't really want me or my daughter to have to take any type of funky medicine or something for research. But I'm okay with doing the saliva samples and stuff. I just don't really want me or my daughter to have to take anything. No one puts anything into our body for research. (P5)

Another participant wanted assurance that MRI scans would stay confidential to avoid incrimination.

Just that maybe not being held against me or being incriminated later to know that anything I did when I used, while I was pregnant, could've affected [their brain]…I wouldn't wanna feel like I'm getting in trouble for trying to help better understand my kid’s body, from what I've been through, you know? (P3)

## Discussion

**Fig. 1:**
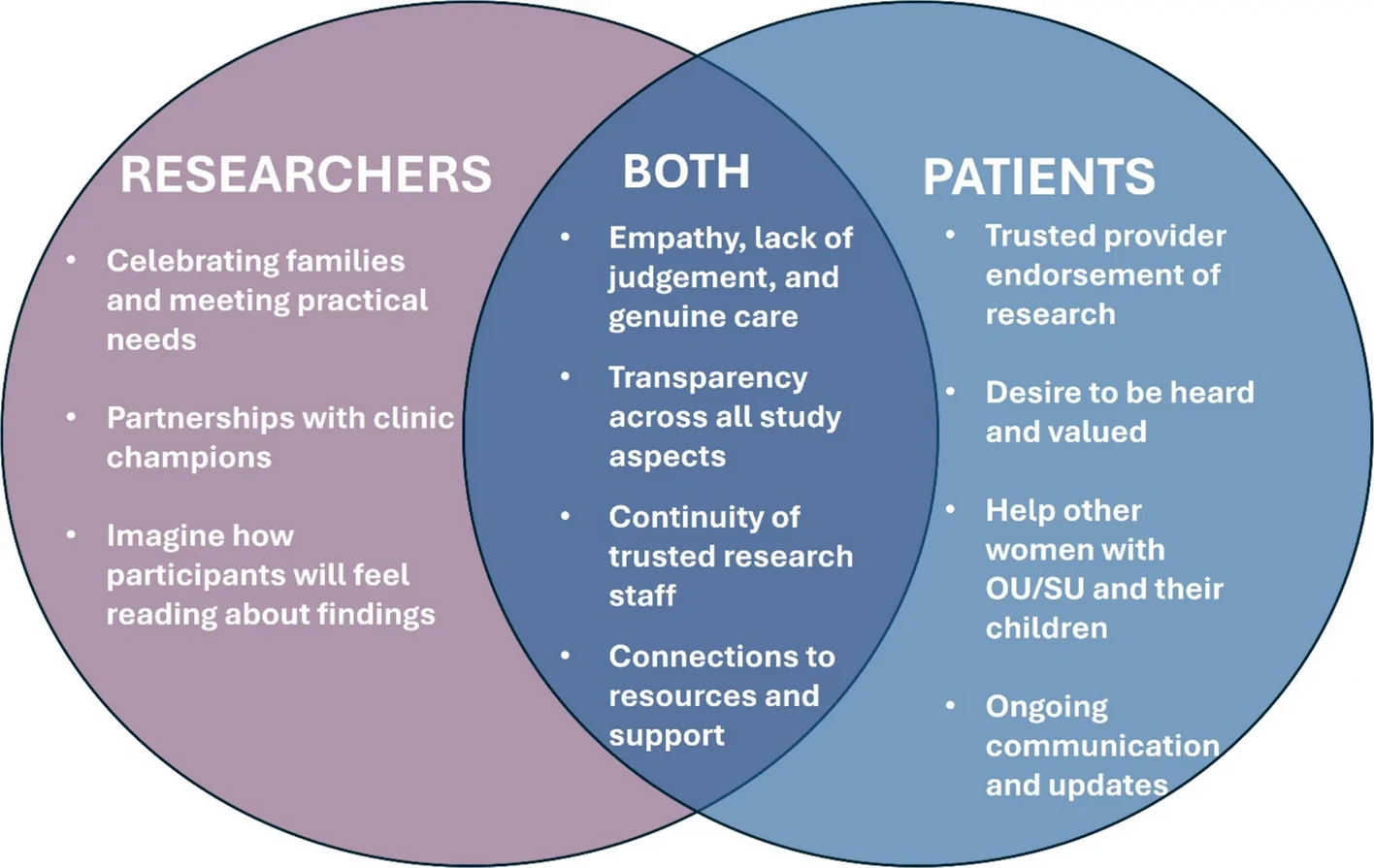
Overlapping and distinct themes from patient (perinatal participants with OUD) and researcher interviews

This qualitative study explored effective strategies for engaging perinatal individuals with OU/SU in longitudinal neurodevelopmental research, informed by insights from participants with lived experience and researchers. Findings highlight shared perspectives on respectful recruitment, engagement, and retention, emphasizing sustained investments of time, effort, flexibility, and funding (see Fig. 1), and have several implications for conducting research with this population. Table 2 summarizes “take home” messages and strategies for implementation.

**Table 2:** Strategies for engaging perinatal participants with OU/SU in neurodevelopmental research

	**Strategies**
Trusting, nonjudgmental relationships	1. Show empathy, lack of judgment, and respect 2. Treat participants as people not data points; value their stories 3. Provide ongoing training to research staff on SU, sensitive engagement of participants 4. Support staff to avoid burnout and promote continuity in team
Recruitment and motivations	1. Align with participant motivations to help other pregnant women with OU/SU 2. Use trusted sources (e.g. prenatal providers, treatment providers, advocates) to recruit, vouch for the study, refer participants 3. Have “boots on the ground” in clinics to form partnerships, identify clinic champions 4. Use social media as a recruitment tool while recognizing limits
Supportive retention	1. Integrate peer support specialists on team 2. Conduct frequent follow-ups, check-ins 3. Provide compensation, transportation, meals, childcare 4. Celebrate family milestones and special days
Flexible and responsive study designs	1. Provide visit flexibility (timing, location) 2. Offer abbreviated protocols when necessary
Transparency	1. Ensure participants understand study goals and procedures 2. Explain mandated reporting with specific examples 3. Provide regular updates and results (both positive and negative) 4. Connect back to participant motivations for joining the study 5. Present results in plain language and disseminate to communities
Neuroimaging and biospecimen protocols	1. Explain purposes of neuroimaging, procedure details, and safety 2. Encourage participants to discuss with their medical providers 3. Talk openly about the process for incidental findings
Ancillary benefits	1. Make meaningful connections to resources with warm handoffs 2. Provide parenting and child development information 3. Offer one-on-one conversations with researchers

### Trusting, nonjudgmental relationships

Both participant groups stressed the importance of creating a research environment centered on trust, care, and connection. Patients’ views of researchers were often shaped by the perceived sincerity and empathy of the research team. Most shared past experiences with prenatal care providers, family and friends that left them feeling ashamed and unseen, which contributed to their hesitancy to engage in research. This is consistent with prior research highlighting stigma and judgment around SU as a significant barrier to research participation [11, 33].

To recruit and retain participants, then, training for research staff should extend beyond technical skills to include comprehensive education on OU/SU and approaches for establishing trust with families. Additionally, strong interpersonal skills, empathy, and a genuine interest in the experiences of participants should be key considerations in hiring staff. Having someone on the research team with lived experience, such as a peer support specialist, can promote trust, help participants navigate the research experience, facilitate connections to services, and serve as a bridge between researchers and participants [33–36]. Hiring peers onto research teams also presents challenges, as some may not meet university educational requirements, lack prior research training, or perform participant-centered work that does not align with standard university schedules and job structures. Continued research is needed to explore how peers can be sustainably integrated onto research teams, including the institutional adaptations required to support their roles and maximize long-term participant retention.

### Recruitment and motivations

Patients and researchers agreed that recruitment through trusted sources, such as medical providers and “clinic champions,” is more effective than flyers or cold calls. Partnerships with treatment facilities and front-line healthcare workers can be instrumental in identifying potential participants, vouching for studies, and supporting retention [37]. When such partnerships are not yet established, building them may require a “boots on the ground” approach, including visiting clinics, compensating providers [38], maintaining communication, and, when feasible, involving partners in study design and implementation [39, 40]. Social media is a cost-efficient, targeted recruitment approach that can be effective for pregnant individuals [10], but while some in the current study agreed, others felt that ads were untrustworthy, underscoring the need for varied recruitment methods [41].

Consistent with prior work [10, 11], patients were motivated to engage in research to advance understanding of prenatal OU, its effects on children, and the resources needed to support impacted families. They felt strongly about helping other pregnant women and breaking the cycle of addiction in their communities, suggesting that recruitment materials should reflect these motivations to foster greater participation.

### Supportive retention

Long-term retention requires ongoing, intentional efforts by researchers amid limited financial and societal support available to participants with OU/SU [42]. Beyond trusting relationships with staff, strategies identified include celebrating milestones, consistent responsiveness to participant needs, and providing logistical supports (e.g. transportation, childcare) during visits. Participants in treatment and early recovery, especially during the perinatal period, may need frequent check-ins between visits for support and reminders. While such supports may be key investments for retention, they are not easy to fit into limited research budgets, which makes it essential for researchers to justify their value to funders to ensure inclusion of these families.

Both groups emphasized staff continuity, particularly for participants with SU who may feel more comfortable with staff already familiar with their backgrounds and who have shown empathy during previous visits. However, intensive study protocols, multiple role demands, and emotional stress can contribute to staff burnout [43], which poses a challenge to maintaining these relationships over time. Ongoing support, realistic expectations, and professional development opportunities can help mitigate staff burnout [44] and promote the stability needed to foster consistent, meaningful participant engagement.

### Flexible and responsive study designs

Researchers must weigh trade-offs when designing protocols for perinatal participants with OU/SU. Patients expressed a strong desire to be “heard” and share their stories, even if beyond the scope of the study questions, but standardized protocols typically require structured formats with limited room for open-ended discussion. Even with a non-judgmental tone, interrupting participants' storytelling to keep interviews concise can still signal a lack of care. Additionally, it may be difficult for some participants, especially those in residential treatment or receiving MOUD, to spend extended time at research sites.

Strategies to address these challenges include developing flexible, abbreviated versions of study protocols and prioritizing the most important assessments for participants who require additional support or have limited availability, and could involve embedding research staff in MOUD and SUD clinics to conduct aspects of the protocol conducive to those settings. Staff can be trained to actively listen and validate meaningful information while gently pivoting back to the protocol. Patients had mixed views on home-based and remote data collection, acknowledging the convenience while noting concerns about impersonality and confidentiality. These findings suggest researchers need to be flexible and consider benefits and limitations of different approaches, balancing the need for collecting reliable, valid data, and accommodating participant preferences to ensure participation of this population longitudinally.

### Transparency

Another commonality across interviews was the desire for transparency throughout the research process. Transparency affirms participants' value, supports autonomy and control, and fosters trust [45]. Patients wanted full information about study procedures and outcomes, while researchers stressed clear communication of study goals, expectations, and legal requirements, especially around sensitive issues such as mandated reporting. Providing concrete examples of reportable behaviors (e.g. child maltreatment) and information on state laws regarding prenatal SU may alleviate uncertainty. Transparency about how results will be used, coupled with ongoing research updates, can prevent breaches of trust that may occur when participants are surprised by how their data are used [46]. Patients expressed eagerness to hear others’ stories and understand how the research makes a difference. To respect participant values [47], findings should be shared in ways that are valuable to participants and communities (e.g. newsletters, personal communication), not solely through academic channels. Involving community members in interpreting results can help ensure insights are grounded in real-world contexts and lead to meaningful, practical policy and practice recommendations [48, 49].

### Neuroimaging and biospecimen collection

Patients discussed their comfort levels and concerns with specific procedures commonly used in neurodevelopmental studies that may be unfamiliar to many participants. It was surprising to find that all were open to participating in infant EEG and MRI and most types of biospecimen collection, which differs from some research showing hesitancy among diverse populations in these methods (e.g. Lee et al.) [6]; almost no one expressed safety concerns. However, their responses also suggest that they had little prior knowledge about the purpose of neuroimaging, how procedures are conducted, or potential risks and benefits (e.g., all believed that MRI involves radiation), echoing findings from a large survey of mothers of infants, in which fewer than half correctly endorsed that MRI does not expose people to radiation, with proportions similar between mothers with and without OU [21]. Research teams are responsible for ensuring informed consent by offering clear information on risks, presenting it in understandable terms, and allowing participants time to consult with their providers regarding the safety of activities. Education on neuroimaging techniques and the collection/use of biospecimens can be provided through visual aids, a virtual tour, or even mock setups to show the equipment and processes firsthand [50].

### Ancillary benefits from research

Finally, both patients and researchers stressed the importance of “giving back” by providing what is sometimes referred to as ancillary care [51]. As one patient suggested, having the opportunity to talk directly with researchers about child development and parenting would be highly valuable. A challenge for researchers is that truly supporting participants in ways that foster long-term engagement often depends on access to treatment and recovery programs (especially that meet the unique needs of parents) – resources that are limited. Additionally, direct, warm handoffs to resources are typically facilitated by full-time social workers who are rarely included in research budgets. While systemic solutions are needed, research sponsors can help by funding efforts to identify resources and ensure warm handoffs that ease participants’ transitions to care.

### Limitations

In line with our findings, we found recruiting patients for this study to be much more challenging than recruiting researchers, particularly when attempting to reach patients without a “warm hand-off” from their provider. Generalizability is constrained by both the small sample and its specificity: women in treatment for OUD living in a large city in a state that does not criminalize prenatal SU. Women living in rural areas or in states with more punitive policies may face different barriers or exhibit greater hesitancy toward participating in research. Furthermore, all participants were engaged in treatment for OUD; their perspectives may differ from women who are not in treatment or are actively using substances. Notably, our study did not uncover some likely barriers to retention, such as postpartum relapse, which can be a common cause of loss to follow-up. Perinatal women face substantial barriers to treatment, including poverty, violence, mental health challenges, competing family roles, interactions with the carceral system, and stigma, and thus the sustained recovery necessary to continue participating in a study.

The researchers interviewed for this study had experience with pregnant women who use substances, however their work was not exclusively focused on OU. As such, findings may reflect perspectives shaped by engagement with diverse forms of perinatal SU, which can differ in associated stigma, criminalization, and research-related barriers. Finally, while the investigators analyzing interviews had training and experience with populations with SU, they were not from within the recovery community. Future studies on this topic would benefit from including individuals with lived experience in study design and interpretation.

## Conclusion

This study reveals that conducting research with perinatal individuals with OU/SU may require departing from traditional ways of conducting research. Focusing on relationships between the research team and participants is particularly important in longitudinal studies with populations with reason to distrust research studies. Greater focus on and funding for ancillary care or facilitated referrals to other sources of care might be needed to strengthen these relationships and improve retention in studies over the longer term. Notably, studies like this that engage individuals with lived experience can also illuminate issues that might be less controversial than would be anticipated, such as collection of biospecimens and the use of neuroimaging. Engaging communities long considered hard to reach may uncover flawed assumptions and new approaches for successful, respectful research.

## Supplementary Information

Supplementary Material 1.

Supplementary Material 2.

Supplementary Material 3.

## Data Availability

Due to the sensitive nature of the qualitative interview data and to protect participant confidentiality, the data are not publicly available. Participants did not provide consent for their transcripts or recordings to be shared beyond the research team.
