# Supplementary material for: Navigating complexity: understanding the experiences, motivations and challenges of conducting research with perinatal individuals who use opioids and other substances
Source: BMC Pregnancy Childbirth. 2026 Jun 8;26:927. doi: 10.1186/s12884-026-09017-0 (PMC13491693; doi:10.1186/s12884-026-09017-0)
Supplement: Supplementary file 1 [file 12884_2026_9017_MOESM1_ESM.docx]

**Appendix A**

**Perinatal Participant (“Patient”) Interview Guide**

Introduction

We are interested in learning about how pregnant women and moms with young children think and feel about participating in a national research study about family health, health behavior and experience and child development, beginning even before birth. The main goals of the study are to look at brain and behavior development that are typical for children, and different factors in children’s experience and environment that may lead to different patterns of development. We want to include families from all different places, cultures, and backgrounds. The long-term purpose of the study is to create programs and services that will help children’s development and can begin as early as possible.

This large study will begin next year and will enroll pregnant women and then follow them and their infants throughout the first 5 years of life. The study will begin during pregnancy to learn about mothers’ experiences and health behaviors, and then will connect these to their infants’ and toddlers’ brain and behavioral development over time. Many research visits with families can occur at their health clinics, their homes, and over the phone or computer. The study will also require mothers and their children to come to [OMITTED FOR BLIND REVIEW] campus 1-2 times per year for research visits and even more often during the first year after their baby is born. There will be many sites across the country doing the same type of research, and the plan is to enroll around 10,000 women. It will be the largest study ever of this type.

The reason I’m talking to you today is because studies are often designed without getting input from the families who will participate. We believe that people who may want to participate should have a “voice” in how these studies are done. We would like to get your input about what would make this planned study engaging and meaningful to families who participate. Our team is really hoping to make their visits as enjoyable as possible, and we want to compensate the moms for their time and effort and also give them information or resources that will be helpful to them as a parent. Since you are [pregnant / a mom of a young child], we are hoping that you can give us some ideas to help us plan for this future study.

During this interview, I will also be asking a couple questions about discrimination, power, and privilege in research. We know in history that researchers treated people poorly and unfairly because of their race, ethnicity, education, income, and disabilities. I would like to hear your thoughts on this but also realize that these are sensitive issues, and issues you probably only talk to with people you really trust. However, too often researchers don’t think about these sorts of things when it comes to our work. Your thoughts and honest responses are really important to us and we’d like to make sure your voice is heard…the good, bad, and ugly…if you are comfortable sharing.

Now I will be asking you questions about participating in research and some different types of activities and procedures that can take place during research visits. There are no right or wrong answers and we value anything you can share with us. As a reminder, your participation is completely voluntary so you can skip any questions you don’t want to answer or stop the interview at any time. Is it ok if I turn on the audio-recorder now so I have an accurate recording of your words and ideas?

**Warm-up:**

1. So before we get started on the research questions, I would love to hear a little bit about [YOUR PREGNANCY / CHILD’s NAME] and how things have been going [FOR YOU / SINCE THEY WERE BORN]? Possible probes: When is your due date? What are you most curious about your baby? What is your child like? What are some things you enjoy doing together?

**Experience with Prenatal Care:**

2. Now I would like to ask about your experience with prenatal care.

2a. What kind of provider [do/did] you see? For example, [do/did] you see an OBGYN, a midwife, a family medicine doctor?

2b. [Do/did] you have any issues in accessing or starting prenatal care?

2c. How much [do/did] you trust your provider? How much [do/did] you feel respected by your provider?

**Research in general:**

3. What are your views about research in general? Do you think your life experience, family or culture has affected these views?

4. Other than talking with me today, have you ever participated in a research study in the past? (For mothers: How about your child?)

4a. IF YES, how was your experience in the other research study(ies)? What did you like about it? What was challenging? What made you decide to participate in this study today?

4b. IF NO, what made you decide to participate in this study today? Have you (or your child) ever been asked to participate in research in the past? Do you know of any other family members or friends who have been in a research study? IF YES, do you know what their experience was like?

4c. What are your views about how researchers treat people? What about people who look like you or come from your community? What about people who did not go to school as long as the researchers? How have these thoughts influenced whether and how you participate in research?

5. (Thinking back to when you were pregnant,) if you and your baby were asked to be in a study that started during pregnancy and lasted until your child was 3-5 years old, and required you to come to [OMITTED] two times a year, is this something you would consider? Why or why not?

5a. Would you be interested in participating in research about opioid use or use of other substances during pregnancy? What would make you feel more comfortable about participating in this type of research? What would make you feel less comfortable?

**Recruitment, Retention and Engagement:**

6. Can you think of anything that would make a long-term study like this more appealing to you? If needed, probe: What could the research team offer to you and your child that would help make the study feel worthwhile to you? What could we provide or tell families that would make them interested? Would it be helpful to provide materials for moms to share with their partner, grandparents or other family members?

7. Now I’m going to talk about some things that our team is considering doing to help moms and their children be more interested and feel connected to a research study. These ideas would be in addition to gift cards that we always give families to compensate them for their time and effort. I am interested to hear what you think about these ideas:

7a. Offering transportation to and from the research visits (e.g. Uber)

7b. Childcare during the visits

7c. Offering to do the study visits remotely when possible

7d. Offering to do the study visits in the family’s home

7e. Information about parenting and child development. If needed, probe: what type of information would be most interesting or helpful?

7f. Feedback about your child’s development. If needed, probe: What type of feedback would be helpful?

7g. Video recordings of you and your child that we make during your visit

7h. A yearly newsletter about the study and what the findings are so far

7i. Can you think of any other ideas that families might enjoy?

8. Another thing that we might do is offer families information about resources in the community for all different needs they may have. These might include community resources for health and mental health needs, substance use, parenting, housing, food, childcare, and education. For example, if a mom was having trouble finding childcare for their baby, we could provide them with a list of childcare providers and phone numbers close to where they live.

8a. Do you think this would be helpful? Why or why not?

8b. What if we had someone on our team who could help moms actually connect with different services? For example, helping a mom find a therapist who is available.

9. We are hoping to invite many pregnant women from all over the [OMITTED] area to participate in this next study and are wondering about the best ways to advertise and spread the word.

9a. Do you have any ideas about how we can reach pregnant moms?

Now I’m going to list a few methods that we’re considering and I’d like you to tell me whether you think it will work and whether you would trust this source.

9b. Flyers about the study posted in your doctor’s office

9c. Your doctor or other medical provider telling you about the study

9d. A member of the research team calling you, texting you, or emailing you

9e. Social media advertisements (probe for specifics).

9f. A friend or family member

9g. Other ideas?

10. For this study we are planning, moms and babies will be coming to [OMITTED] for a lot of research visits and we really want to have staff who will make families feel most comfortable be leading visits with families. Are there any important characteristics or qualities that you think would be important for the research staff to have? If needed, probe: For example, would it matter if they are women or men? Someone older or younger? Someone who is good at playing with babies? Someone who is from your community? If needed, probe: if you think about other people like doctors or teachers, what kinds of things make you like them and trust them?

**Concerns about sharing information**

I’d now like to ask you some questions about information you may have shared with your prenatal care provider, and laws on substance use that you might have concerns about.

14. When you (are/were) receiving prenatal care, (is/was) there anything that you didn’t want to tell or share with your provider?

14a. (Do/Did) you ever feel like you had to modify, change, or hide what you (tell/told) your provider? Why or why not?

14b. What things (does/did) your provider to do make you feel more comfortable or less comfortable?

15. Do/Did you share information with your provider about using substances, such as opioids, marijuana, alcohol, nicotine, or other drugs? Why or why not?

15a. Tell us about your comfort level in sharing information about substance use. What (does/did) your provider do to make you more or less comfortable?

15b. Who (is/was) your most trusted person to talk with about opioid use during your pregnancy?

15c. Do you ever avoid getting treatment from a provider because you were worried about them finding out about substance use? Do you ever avoid getting care that you feel is urgent or important?

16. Please tell us what you know about the law is in [OMITTED] about substance use during pregnancy?

16a. How did you learn about this law?

16b. What do you think about the law?

16c. If you’re not familiar with the law, what do you think that it says about opioid use or use of other substances in pregnancy? If unsure: Do you think substance use in pregnancy is a criminal offense or could lead to a child being removed from their mother’s custody?

**Acceptability of Assessment Procedures**

11. Now I’m going move to some questions about assessments that we often do with babies, young children and parents in our research. We do these assessments to learn more about healthy brain development in children. You might not have heard of these before or know much about them and that’s ok. The first one is called an EEG.

11a. Have you ever heard of an EEG? Do you know anything about it?

11b. Based on what you know, do you think that it is safe for babies? Safe for children? Safe for pregnant women?

11c. EEG is a way of recording the normal activity in the brain by putting sensors on a person’s head. Babies or kids who are doing an EEG wear a stretchy hat that holds the sensors. Each sensor has a little sponge that holds a bit of salt water, which touches their head over their hair. During the EEG, the child watches a short kid-friendly video or listens to some speech sounds. The cap doesn’t hurt at all, and we can stop or take a break if the child is upset for any reason. The whole thing takes about 30-40 minutes. With this information, how likely would you be to let your newborn/infant/toddler participate in an EEG? What concerns would you have, if any?

12. The other assessment I want to ask you about is called an MRI.

12a. Have you ever heard of an MRI? Do you know anything about it?

12b. Based on what you know, do you think it is safe for babies? Safe for children? Safe for pregnant women?

12c. Do you know if having an MRI exposes a person to radiation? Yes, no, not sure?

12d. MRI is a way of taking different types of pictures of parts of the body. We use it to take pictures of the brain. The MRI machine is a big tube that a person lays down in. It uses strong magnets to take the pictures. The MRI makes a lot of noise, so people wear earplugs while inside. For babies and young kids, we do a “lullaby MRI”, which means we have them fall asleep like they would at home, then go into the MRI to take the pictures. A researcher or their parent is next to the MRI with them the whole time. There is no radiation from MRI, like there is with X-Rays, and MRI is safe for people of all ages. With this information, how likely would you be to let your newborn/infant/toddler participate in an MRI? What concerns would you have, if any?

13. In some studies, researchers collect biospecimen samples from moms and their babies. These might include a blood draw, a saliva sample, hair or nail clippings, or urine or stool from the baby’s diaper. The reason researchers collect these samples is to learn about the mind-body connection and how are bodies are tied to our life experiences. Do you think you would let researchers collect samples like these from you or your baby? Why or why not? (If needed, probe about whether certain types of samples would be more acceptable, and any circumstances that would make sample collection more acceptable).

**Closing**

Those are all the questions that I have. Is there anything else you’d like to share before we finish up? Do you have any questions for me? Thank you so much for sharing your insights and experiences with me today!
