# Supplementary material for: Navigating complexity: understanding the experiences, motivations and challenges of conducting research with perinatal individuals who use opioids and other substances
Source: BMC Pregnancy Childbirth. 2026 Jun 8;26:927. doi: 10.1186/s12884-026-09017-0 (PMC13491693; doi:10.1186/s12884-026-09017-0)
Supplement: Supplementary file 2 [file 12884_2026_9017_MOESM2_ESM.docx]

**Appendix B**

**Researcher Interview Guide**

Introduction

Hello. My name is [X] and I would like to invite you to participate in a survey about how laws regarding substance use during pregnancy affect pregnant women and their children. I am a part of a larger study team, and our goal is to learn more about how to make laws more compassionate and able to protect pregnant women and their infants. We would like to interview you because of your experience conducting research on prenatal exposure to drugs or alcohol. Your participation in this study is voluntary and you can stop at any time. We are providing compensation of $50 for your time and effort.

1. First, do you know what the law is in your state about substance use during pregnancy?

- *How did you learn about it?*
- *What do you think about the law?*
- *Have you ever interacted with the criminal justice or child protection systems in relation to your research?*

2. Have you ever made modifications to your study design in order to enroll substance-using pregnant women in your research?

- *If so, what kind of modifications have you made?*
- *Did these modifications compromise anything else important about the study (for example, statistical power or feasibility)?*
- *Did you choose to make these modifications yourself or were they recommended by your IRB or institution?*

3. What are some of the approaches you or others have taken in conducting this research that you would recommend to other researchers as best practices?

- *Were there any groups you consulted with in advance of conducting the research?*
- *Were there ways to avoid reporting requirements that helped conduct your study?*
- *Were there approaches you took that helped pregnant women feel more comfortable participating in your study?*
- *Are there any approaches you would not recommend?*

4. If you could design an ideal law regarding substance use during pregnancy, what would it say?

- *Would the law require reporting by clinicians or researchers?*
- *Would it require preferential access to treatment programs for pregnant women?*
- *Would substance use during pregnancy be considered a type of child abuse?*
- *Are there any protections it should have that are not provided by a Certificate of Confidentiality?*

5. Is there any research that you think is needed on substance use during pregnancy that might not be happening because of these legal requirements?
6. Have you had any interactions with the criminal justice system or child protective services related to your research?

- *Have they been positive or negative?*
- *Have they requested information from your study?*
- *Did you have a certificate of confidentiality? If so, was it a helpful protection?*

7. Is there anything else you would like to share with us?

Thank you for your time and willingness to talk with us. We truly appreciate the information you have provided us, and will work to disseminate this information to develop better practices and policies to help pregnant women using opioids and their infants.
